# P300 event-related potentials in patients with different subtypes of depressive disorders

**DOI:** 10.3389/fpsyt.2022.1021365

**Published:** 2023-01-13

**Authors:** Yun Wang, Canxin Li, Xiaohua Liu, Daihui Peng, Yan Wu, Yiru Fang

**Affiliations:** ^1^Division of Mood Disorders, Shanghai Mental Health Center, Shanghai Jiao Tong University School of Medicine, Shanghai, China; ^2^The Third People’s Hospital of Foshan, Foshan Mental Health Center, Foshan, Guangdong, China; ^3^Department of Psychiatry, Shanghai Mental Health Center, Shanghai Jiao Tong University School of Medicine, Shanghai, China; ^4^Shanghai Key Laboratory of Psychotic Disorders, Shanghai Mental Health Center, Shanghai Jiao Tong University School of Medicine, Shanghai, China; ^5^CAS Center for Excellence in Brain Science and Intelligence Technology, Shanghai, China

**Keywords:** major depressive disorders, diagnosis, P300, event-related potentials (ERPs), subtype

## Abstract

**Objective:**

To explore the differences in event-related potentials (ERPs) of the subclinical types of major depressive disorders (MDD): melancholic (MEL), atypical (ATY), and anxious (ANX).

**Methods:**

Patients with MDD treated in the Clinical Department of Shanghai Mental Health Center between September 2017 and December 2020 were prospectively included. This study was approved by the Ethics Committee of the Shanghai Mental Health Center. They were evaluated using the Mini-International Neuropsychiatric Interview (MINI), 17-item Hamilton Depression Scale (HAMD-17), 30-item Self-rated Inventory of Depressive Symptomatology (IDS-30SR), 16-item Quick Inventory of Negative Symptom Scale (QIDS-16SR), and auditory and visual P300 ERPs.

**Results:**

Finally, 27, 14, and 20 patients with MEL, ATY, and ANX MDD were included in this study, respectively. There were no significant differences in demographic characteristics and HAMD-17, IDS-30SR, and QIDS-16SR total scores among the three groups (all *P* > 0.05). On the C3 lead, the latency for patients with MEL MDD was the longest, and the latency for patients with ATY MDD was the shortest (MEL vs. ATY vs. ANX: 373.89 ± 6.60 vs. 344.79 ± 9.78 vs. 359.33 ± 7.62, *P* = 0.039). On the Pz lead, the latency for patients with MEL MDD was the longest, and the latency for patients with ATY MDD was the shortest (MEL vs. ATY vs. ANX: 376.14 ± 6.51 vs. 347.21 ± 9.42 vs. 362.22 ± 8.63, *P* = 0.047). There were no differences in visual P300 ERPs among the three groups.

**Conclusion:**

There are significant differences in auditory C3 and Pz latency among MEL, ATY, and ANX MDD. These differences could help diagnose the subtype of MDD.

## Background

Major depressive disorder (MDD) is a common and well-researched type of depressive disorder that is characterized by a persistent low mood, a lack of positive affect, and a loss of interest in usually pleasurable activities (anhedonia) that is different from the patient’s usual self and causes significant distress or impairment for ≥2 weeks (1, 2). MDD has a heterogeneous clinical presentation such that two patients with the diagnosis may have only a few symptoms in common (3). The worldwide prevalence of MDD is approximately 6% per year, with a lifetime prevalence of 20% (4). MDD classification by episode (first or recurrent), status (such as partial or full remission), and severity are relevant to treatment (mild, moderate, and severe) (3–5). The reported risk factors for MDD include a family or personal history of major depression and/or substance abuse, chronic medical illness, alcohol and substance use, stressful life events including loss (including bereavement or divorce), major life changes such as job change or financial difficulty, domestic abuse or violence, female sex, low income and unemployment, and disability (2, 4). The prognosis for MDD is variable (2, 4). It is unremitting in about 15% of patients and recurrent in about 35%, with the risk of recurrence increasing with each additional episode of major depression (2, 4).

Some patients have specific subtypes of depression, including melancholic (MEL), atypical (ATY), and anxious (ANX) depressive disorders, which may be clinically useful for predicting outcomes and choosing treatment (3–5). Discrimination among the different types of MDD is often difficult as the subtypes can have overlapping features. Still, the different subtypes of depression involve different mechanisms. For example, MEL MDD involves hypothalamic-pituitary-adrenal (HPA) axis hyperactivity, while ATY MDD involves HPA hypoactivity. Therefore, such differences could be quantified to help diagnose the disease and guide management (6).

Event-related potentials (ERPs) are measures of the brain’s neural activity, displaying excellent temporal resolution and possibly being used to examine the abnormalities associated with MDD (7–9). Indeed, MDD is characterized by reduced P300 ERPs (8), representing a latency in cognitive processes, including memory and related constructs (10). MDD is also associated with reduced late positive potentials (LPPs) in response to negative and positive stimuli (11).

Event-related potentials could be used to distinguish among MDD subtypes. Some studies examined the ERP features in MEL MDD (12–20), but the sample size could be as small as seven patients (19), or studies could include a mixture of non-MEL MDD and healthy individuals in the control group (12, 14, 17–20) or only healthy controls (13, 15, 16). In addition, these previous studies did not consider ATY MDD and ANX MDD. There is a lack of data for the direct comparison of ERPs among MEL MDD, ATY MDD, and ANX MDD.

Therefore, this study aimed to explore the differences in ERP of the subclinical types of MDD: MEL, ATY, and ANX. Since ERPs are easily measured, they could be cost-effective parameters for diagnosing and managing MDDs.

## Materials and methods

### Study design and patients

In this study, patients with MDD treated in the Clinical Department of Shanghai Mental Health Center between September 2017 and December 2020 were prospectively included. This study was approved by the Ethics Committee of the Shanghai Mental Health Center. Written informed consents were obtained from all participants.

The inclusion criteria for patients with MDD were (1) met the diagnostic criteria of depressive episode described in the Diagnostic and Statistical Manual of Mental Disorders 5th edition (DSM-5), (2) 17-item Hamilton Depression Scale (HAMD-17) score ≥ 17, (3) Han Chinese of 18–60 years of age and right-handed, (4) first depressive episode or did not receive anti-depressive therapy within the last 4 weeks, and (5) did not receive any anti-depressive drugs, physical treatment, or mental therapy within the past year. The exclusion criteria were (1) history of mania or hypomanic episode, (2) psychoactive substances or alcohol-induced mental disturbance, organic disease-induced mental disturbance, or other mental diseases, (3) pregnant or breastfeeding women or planning for pregnancy, (4) with serious ideation or behaviors of suicide, or (5) severe somatic diseases or autoimmune diseases.

### Assessment

The Chinese version of the Mini-International Neuropsychiatric Interview (MINI) (21) was used in this study to screen the patients before inclusion.

The HAMD-17 and 30-item Self-rated Inventory of Depressive Symptomatology (IDS-30SR) were used to assess the clinical characteristics of patients. The HAMD-17 included 17 items, and the total score is categorized into mild (7–17 points), moderate (18–24 points), and severe (>24 points), and patients with a total score of <7 points were considered with no evident depressive symptoms. In this study, the four-level HAMD-17 model was used, which included core depressed mood (HAMD items 1, 7, and 8), somatic anxiety (HAMD items 4–6, 11–13, and 15), psychic anxiety (HAMD items 2, 9, 10, and 17), and loss of appetite (HAMD items 12 and 16) (22).

The IDS-30SR is also commonly used in the studies of depressive disorders. In contrast to HAMD-17, each question in IDS-30RS is rated from 0 to 3 points, and higher scores indicate more severe depression-related symptoms. A total score of IDS-30SR, calculated by adding up the scores of all 30 questions, >18 points indicates the presence of evident depression symptoms. In this study, IDS-30SR was used for the classification of depressive disorders of patients on inclusion, and the three-level model (dimensions of depression/emotional, anxiety/somatic, and sleep disorders) was used to explore the clinical characteristics of patients, i.e., emotion/cognition (IDS items 5, 8, 10, 11/12, 15–18, 20, 22, and 29), anxiety/somatization (IDS items 6, 23–28, and 30), and sleep (IDS items 1–4) (23).

The 16-item Quick Inventory of Negative Symptom Scale (QIDS-16SR) was used using the four-level scoring method. The score of each item was 0–3 points, and the scale’s total score was 27 points. Higher scores indicated heavier symptoms. The total score was calculated using the highest score of questions 1–4, 6–9, and 15–16 plus the scores of the other items. A score of 1–5 indicated no depression, 6–10 indicated mild depression, 11–15 indicated moderate depression, 16–20 indicated severe depression, and 21–27 indicated very serious depression (24).

The six-item quality of life scale (qol-6) was compiled by the clinician and used to measure the overall quality of life of patients in the past month, with a total of six questions. The scale adopted the five-grade scoring method, 1 = very poor, 2 = poor, 3 = average, 4 = good, and 5 = very good. A total score of QOL <18 represented a poor quality of life.

### Recording of ERPs

In this study, ERPs were recorded for all the included subjects using the 32-lead electrode cap. A BrainAmp MR Plus (Brain Vision Solutions, Montreal, QC, Canada) was used to record the electroencephalogram (EEG). During the measurement, the subjects were asked to sit on a chair, keep quiet, and wear noise-canceling headphones. All measurements were performed by the same professional. The examinations were performed approximately 3 h after a meal. The scalp was washed before EEG to the scalp’s resistance at <5,000 Ω. The relevant matters needing attention included that the examination had to remain painless, the patients needed to relax and keep quiet during the procedures, the patients needed to try reducing the frequency of blinking, and the investigators in the EEG room needed to inform the patients before the uninterruptable procedures started.

The electrodes were set according to the international standard 10- to 12-lead system. The points, including Fpz, Fz, Cz, Pz, and Oz, were set from anterior to posterior along the sagittal line. The distance from Fpz to the root of the nose and the distance from Oz to the external occipital protuberance accounted for 10% of the total length of the line, while the other points were separated by 20% of the total length of the line. The points, including T3, T4, C3, and C4, were set from left to right along the coronal line. The distance from T3 or T4 to preauricular points accounted for 10% of the total length of the line, while the other points (including Cz) were separated by 20% of the total length of the line. The points on the lateral view included Fp1, Fp2, F7, F8, T5, T6, O1, and O2. The distance from Fp1 or Fp2 to Fpz, as well as the distance from O1 or O2 to Oz, accounted for 10% of the total length of the line, while the other points (including T3 and T3) were separated by 20% of the total length of the line. The other electrodes included F3 and F4, at the center between Fp1 and Fp2, and C3 and C4, respectively. The electrodes P3 and P4 were at the center between C3 and C4, as well as O1 and O2, respectively. The auditory evoked P300 and visual evoked P300 were used to acquire the amplitude and latency of ERP.

### Data collection

The general demographic data of the patients were collected, including name, age, sex, education duration, occupation status, marital status, HAMD-17, IDS-30S scores, QIDS-16SR scores, QOL-6 scores, and ERP data.

### Statistical analysis

SPSS 21.0 (IBM, Armonk, NY, USA) was used for statistical analysis. All continuous data were described using means ± standard deviations. The Kolmogorov-Smirnov test was used for the normality test. One-way analysis of variances (ANOVA) was used to compare data among three or more groups. The chi-square test was used for the comparison of categorical data. The Analyzer v2.01 software was used to analyze EEG and ERP data. The band-pass filter for the tasks of auditory P300 and visual P300 was 0.05–30 Hz, and the block duration was 900 ms. The duration before stimulus presentation was 100 ms, and the duration after stimulus presentation was 900 ms. For the baseline correction, the first 100 ms of the stimulation was used as the reference, and the EEG artifact was adjusted. The segments with the peak and trough of amplitude >100 μV were considered artifacts. All statistical analyses were two-sided, and *P*-values < 0.05 were considered statistically significant.

## Results

### Characteristics of the patients

Finally, 27, 14, and 20 patients with MEL, ATY, and ANX MDD were included in this study, respectively. The patients with MEL MDD included eight males (29.6%) and 19 females (70.4%), mean age was 29.7 ± 1.2 years, and mean education duration was 15.1 ± 0.5 years. The 14 patients with ATY MDD included six males (42.9%) and eight females (57.1%), mean age was 26.0 ± 2.6 years, and mean education duration was 15.9 ± 0.3 years. The 20 patients with ANX MDD included nine males (45%) and 11 females (55%), mean age was 28.4 ± 1.9 years, and mean education duration was 15.1 ± 0.3 years. The sex, age, education duration, height, body weight, occupation status, and marital status were not significantly different among the three groups (all *P* > 0.05) (Table 1).

**TABLE 1:** Comparison of the baseline characteristics.

General characteristics	MEL (*n* = 27)	ATY (14)	ANX (20)	*P*
Sex (M/F)	8/19	6/8	9/11	0.506
Age (years)	29.7 ± 1.2	26.0 ± 2.6	28.4 ± 1.9	0.351
Education duration (years)	15.1 ± 0.5	15.9 ± 0.3	15.1 ± 0.3	0.356
Height (cm)	165.8 ± 1.5	167.2 ± 1.8	169.7 ± 2.2	0.289
Body weight (kg)	57.1 ± 1.8	58.5 ± 2.0	59.1 ± 2.3	0.743
Occupation status	–	–	–	0.346
Professional	17	5	9	–
Retired	0	1	0	–
Students	8	5	8	–
Unemployed	2	3	3	–
Marital status	–	–	–	0.707
Unmarried	16	11	15	–
Married/living together	7	2	3	–
Divorced/separated	4	1	2	–
Age at the first episode (years)	25.8 ± 1.7	22.7 ± 2.3	22.9 ± 1.8	0.392
Duration of this episode (weeks)	9.7 ± 2.3	13.6 ± 6.8	9.7 ± 4.0	0.765
Total disease duration (weeks)	29.9 ± 6.7	26.6 ± 11.4	33.4 ± 9.5	0.896
First episode	14	7	11	–
QOL	15.2 ± 0.6	16.9 ± 0.6	16.1 ± 0.7	0.726
Physical condition	2.5 ± 0.8	2.5 ± 0.8	2.7 ± 0.2	0.513
Psychological conditions	1.8 ± 0.1	2.2 ± 0.2	1.9 ± 0.2	0.253
Economic status	2.7 ± 0.2	3.3 ± 0.2	2.9 ± 0.1	0.057
Working condition	2.2 ± 0.2	2.3 ± 0.2	2.4 ± 0.2	0.737
Relationship with the family	3.0 ± 0.2	3.2 ± 0.3	3.1 ± 0.2	0.804
Relationship with others	3.0 ± 0.1	3.0 ± 0.2	3.1 ± 0.1	0.726

MEL, melancholic; ATY, atypical; ANX, anxious; QOL, quality of life.

### Psychometric scores

The total HAMD-17 score in the ANX group (24.3 ± 1.1) was higher than in the MEL group (23.9 ± 1.1) and ATY group (21.9 ± 0.7). The total IDS-30SR score in the MEL group (45.6 ± 2.5) was higher than in the ATY group (42.3 ± 2.6) and ANX group (38.9 ± 2.2). The total QIDS-16 score in the MEL group (15.9 ± 1.3) was also higher than in the ATY group (13.0 ± 1.4) and ANX group (13.9 ± 0.8). The differences in the dimensions of sleep (F = 4.064, *P* = 0.022), somatic anxiety (F = 10.562, *P* < 0.001), and cognitive disorder (F = 4.852, *P* = 0.011) in HAMD-17 were significantly different among the three groups, while the total score and scores of other dimensions were not significantly different. Of the IDS-30SR subscores, the difference in emotion/cognition was significant among the three groups (F = 6.680, *P* = 0.003), while the total score and scores of other dimensions were not statistically significant among the three groups. The QIDS-16 total scores and subscores were not significantly different among the three groups (all *P* > 0.05) (Table 2).

**TABLE 2:** Comparison of the clinical characteristics of the patients.

Scale	MEL	ATY	ANX	*P*
**HAMD-17**				
Emotion	9.26 ± 0.34	9.62 ± 0.43	8.53 ± 0.48	0.220
Sleep	4.19 ± 0.30	2.77 ± 0.47	3.05 ± 0.43	**0.022**
Somatic anxiety	5.52 ± 0.43	4.00 ± 0.49	7.37 ± 0.45	**<0.001**
Psychic anxiety	3.89 ± 0.21	3.57 ± 0.21	4.26 ± 0.35	0.297
Cognitive disorder	1.37 ± 0.27	0.77 ± 0.30	2.05 ± 0.16	**0.011**
Total HAMD score	23.93 ± 1.07	21.92 ± 0.74	24.26 ± 1.14	0.373
**IDS-30SR**				
Emotion/cognition	20.48 ± 0.86	15.46 ± 1.47	15.26 ± 1.39	**0.003**
Anxiety	11.26 ± 0.77	9.31 ± 0.58	10.05 ± 0.74	0.210
Sleep	5.89 ± 0.42	5.46 ± 0.53	4.37 ± 0.47	0.055
Total IDS score	45.63 ± 2.54	42.31 ± 2.61	38.90 ± 2.23	0.151
**QIDS-16**				
Sleep disturbance	2.41 ± 0.17	2.15 ± 0.27	1.84 ± 0.23	0.149
Sad mood	2.16 ± 0.18	1.73 ± 0.27	1.94 ± 0.20	0.380
Appetite and weight	1.63 ± 0.21	1.23 ± 0.32	1.68 ± 0.24	0.468
Concentration	1.84 ± 0.15	1.67 ± 0.28	1.41 ± 0.21	0.279
Self-outlook	1.96 ± 0.22	1.83 ± 0.24	1.63 ± 0.29	0.454
Suicidal ideation	1.08 ± 0.16	0.75 ± 0.22	1.12 ± 0.23	0.454
Involvement	2.20 ± 0.17	1.58 ± 0.19	1.88 ± 0.23	0.115
Energy fatigue	1.84 ± 0.16	1.42 ± 0.19	1.41 ± 0.19	0.140
Psychomotor anxiety	1.56 ± 0.16	1.46 ± 0.24	1.37 ± 0.21	0.772
Total QIDS score	15.85 ± 1.25	13.00 ± 1.35	13.94 ± 0.79	0.323

MEL, melancholic; ATY, atypical; ANX, anxious; IDS-30SR, 30-item inventory of depressive symptomatology; HAMD-17, 17-item hamilton depression scale; QIDS-16, 16-item quick inventory of negative symptom scale. Bold values represent the *P* < 0.05, there was statistical difference.

### Auditory P300 ERPs

For the auditory P300 task, the leads including C3, C4, P3, P4, Cz, Pz, CP1, CP2, and POz were used. On the C3 lead, the latency for patients with MEL MDD was the longest, the latency for patients with ATY MDD was the shortest, and the difference among the three groups was statistically significant (MEL vs. ATY vs. ANX: 373.89 ± 6.60 vs. 344.79 ± 9.78 vs. 359.33 ± 7.62, F = 3.433, *P* = 0.039). On the Pz lead, the latency for patients with MEL MDD was the longest, the latency for patients with ATY MDD was the shortest, and the difference among the three groups was statistically significant (MEL vs. ATY vs. ANX: 376.14 ± 6.51 vs. 347.21 ± 9.42 vs. 362.22 ± 8.63, *P* = 0.047) (Table 3). In the auditory P300 task, the differences in amplitude on all the leads were not statistically significant among the MEL, ATY, and ANX groups (all *P* > 0.05) (Table 4).

**TABLE 3:** Comparison of latency of baseline auditory P300 task.

Lead	MEL	ATY	ANX	*P*
C3	373.89 ± 6.60	344.79 ± 9.78	359.33 ± 7.62	**0.039**
C4	369.07 ± 7.40	345.86 ± 9.01	359.56 ± 8.42	0.166
P3	373.75 ± 6.99	350.14 ± 9.57	366.00 ± 7.66	0.136
P4	375.54 ± 6.51	350.71 ± 11.2	363.61 ± 8.61	0.124
Cz	361.39 ± 6.78	346.93 ± 8.41	356.94 ± 7.31	0.425
Pz	376.14 ± 6.51	347.21 ± 9.42	362.22 ± 8.63	**0.047**
CP1	369.14 ± 7.73	346.14 ± 9.46	360.67 ± 7.85	0.183
CP2	371.18 ± 7.57	354.43 ± 10.20	356.89 ± 7.52	0.286
POz	380.86 ± 6.60	365.86 ± 10.20	358.89 ± 9.41	0.136

MEL, melancholic; ATY, atypical; ANX, anxious. Bold values represent the *P* < 0.05, there was statistical difference.

**TABLE 4:** Comparison of amplitude of baseline auditory P300 task.

Lead	MEL	ATY	ANX	*P*
C3	8.11 ± 0.93	8.60 ± 1.20	10.93 ± 1.05	0.139
C4	8.87 ± 1.00	9.76 ± 1.57	10.37 ± 1.43	0.676
P3	12.07 ± 1.14	11.82 ± 1.58	13.08 ± 0.93	0.771
P4	11.95 ± 1.23	10.57 ± 1.66	13.26 ± 0.93	0.435
Cz	9.58 ± 1.14	10.83 ± 1.57	12.7 ± 1.69	0.275
Pz	13.49 ± 1.20	12.43 ± 1.56	15.09 ± 1.22	0.441
CP1	10.75 ± 1.12	11.73 ± 1.42	13.53 ± 1.28	0.274
CP2	11.62 ± 1.17	11.64 ± 1.44	13.19 ± 1.46	0.655
POz	12.04 ± 1.31	10.90 ± 1.86	12.84 ± 1.10	0.694

MEL, melancholic; ATY, atypical; ANX, anxious.

### Visual P300 ERPs

For the visual P300 task, the leads including C3, C4, P3, P4, Cz, Pz, CP1, CP2, and POz were used. The latencies for patients among the three groups were not significantly different (all *P* > 0.05) (Table 5). The amplitude differences were also not significantly different among the three groups (all *P* > 0.05) (Table 6).

**TABLE 5:** Comparison of latency of baseline visual P300 task.

Lead	MEL	ATY	ANX	*P*
C3	410.89 ± 4.37	410.08 ± 7.81	406.17 ± 6.40	0.823
C4	417.46 ± 3.65	397.46 ± 9.38	405.61 ± 6.96	0.066
P3	408.96 ± 5.21	401.77 ± 8.00	400.72 ± 8.51	0.621
P4	408.46 ± 5.16	398.00 + 8.93	386.5 ± 9.32	0.090
Cz	411.29 ± 3.91	392.54 ± 8.48	408.5 ± 5.32	0.064
Pz	411.11 ± 4.94	396.46 ± 9.27	401.67 ± 8.33	0.323
CP1	412.43 ± 4.57	394.38 ± 8.66	404.61 ± 7.21	0.159
CP2	411.18 ± 4.61	393.38 ± 8.36	407.39 ± 6.76	0.152
POz	410.46 ± 5.27	392.69 ± 8.30	391.78 ± 8.68	0.090

MEL, melancholic; ATY, atypical; ANX, anxious.

**TABLE 6:** Comparison of amplitude of baseline visual P300 task.

Lead	MEL	ATY	ANX	*P*
C3	12.89 ± 1.241	15.1 ± 1.859	11.63 ± 1.405	0.337
C4	12.79 ± 1.215	13.38 ± 2.465	11.38 ± 1.717	0.719
P3	12.98 ± 1.52	14.92 ± 1.923	12.41 ± 1.642	0.638
P4	11.66 ± 1.506	14.77 ± 1.884	10.85 ± 1.733	0.337
Cz	15.45 ± 1.466	16.49 ± 2.967	14.37 ± 1.674	0.779
Pz	14.68 ± 1.469	16.7 ± 2.178	13.83 ± 1.705	0.582
CP1	14.96 ± 1.419	15.55 ± 2.614	13.99 ± 1.698	0.852
CP2	14.5 ± 1.493	15.66 ± 2.384	14.09 ± 1.623	0.851
POz	12.71 ± 1.473	15.2 ± 1.926	11.29 ± 1.688	0.357

MEL, melancholic; ATY, atypical; ANX, anxious.

## Discussion

The results suggest significant differences in auditory C3 and Pz latency among MEL, ATY, and ANX MDD, but without differences in auditory amplitude, visual latency, or visual amplitude. These differences could help diagnose the subtype of MDD.

The studies of ERPs in MDD are limited, especially in sample size or control groups (12–20). In addition, they examined MEL MDD vs. non-MEL individuals. In addition, several studies are from the 1980s and 1990s, and various ERP parameters were examined. Two previous studies examined ERP components indicating preparatory activity prior to a behavior (16, 19). Khanna et al. (16) found lower BP amplitude in MEL MDD compared with healthy controls, while Elton et al. (19) observed no differences among MEL MDD, reactive MDD, and healthy controls.

In the present study, the auditory C3 and Pz latencies were the longest for MEL MDD and the shortest for ATY MDD. These differences between MEL MDD and ATY MDD could be due to the opposite HPA involvement in the two conditions: MEL MDD involves HPA axis hyperactivity, while ATY MDD involves HPA hypoactivity (6). A recent study also showed significant alterations in brain structure in patients with MEL MDD (25). Gangadhar et al. (13) showed smaller auditory P300 amplitudes in MEL MDD compared with healthy controls, without differences in latencies. Quinn et al. (20) reported no differences in auditory P300 latencies or amplitudes between MEL MDD and controls. On the other hand, Kerr et al. (14) showed longer auditory P300 latencies in MEL MDD compared with non-MEL MDD, supporting the present study. Still, a study revealed significant heterogeneity in multimodal neuroimaging within the MEL MDD subtype, indicating that work is still required to define the MDD subtypes adequately (26).

In the present study, there were no differences in visual P300 latencies and amplitudes among the three groups. A study suggested differences in visual P300 amplitudes in patients with clinical high-risk vs. healthy controls, but the patients were not formally diagnosed with MDD. An early study showed differences in visual P300 amplitudes between patients with MDD and healthy controls (27).

Nevertheless, the determination of the ERPs in patients with MDD could be clinically significant for predicting treatment efficacy. Indeed, Lee et al. (28) showed that patients with low frontal alpha asymmetry had a better treatment efficacy than those with high asymmetry. The present study included only untreated patients and had no multiple measurements in time. Future studies should examine the ERPs before and after treatment in different MDD subtypes.

This study had limitations. It was a single-center study, and the sample size was limited. In addition, the sample size was too small for correlation or multivariable analysis. Although the study was prospective, its cross-sectional design prevented the analysis of cause-to-effect relationships. No healthy controls were included. More multicenter, controlled trials with larger sample sizes are needed to provide higher-grade evidence.

In conclusion, there are significant differences in auditory C3 and Pz latency among MEL, ATY, and ANX MDD, but without differences in auditory amplitude, visual latency, or visual amplitude. These differences could help diagnose the subtype of MDD.

## Data availability statement

The original contributions presented in this study are included in the article/supplementary material, further inquiries can be directed to the corresponding authors.

## Ethics statement

The studies involving human participants were reviewed and approved by Ethics Committee of Shanghai Mental Health Center. The patients/participants provided their written informed consent to participate in this study.

## Author contributions

YWa, XL, DP, YWu, and YF: conception and design of study and acquisition of data. All authors contributed to analysis and interpretation of data and approved the submitted version.
